# Individual-level HPV vaccination verification in Botswana: lessons learned from implementation of the HOPE II trial

**DOI:** 10.3389/fpubh.2026.1821335

**Published:** 2026-07-01

**Authors:** Swetha Bindu Jammalamadugu, Lesedi Tirelo, Nabila Youssouf, Grace Umutesi, Meighan Leta Krows, Lesego Kuate, Keatlaretse Siamisang, Ruanne V. Barnabas, Rebecca Luckett

**Affiliations:** 1 Botswana Harvard Health Partnership, Gaborone, Botswana; 2 London School of Hygiene and Tropical Medicine, London, United Kingdom; 3 Department of Global Health, University of Washington, Seattle, WA, United States; 4 Division of Infectious Diseases, Mass General Brigham, Boston, MA, United States; 5 Ministry of Health, Gaborone, Botswana; 6 Harvard Medical School, Boston, MA, United States; 7 Department of Obstetrics and Gynecology, Beth Israel Deaconess Medical Center, Boston, MA, United States; 8 Department of Obstetrics and Gynecology, University of Botswana, Gaborone, Botswana

**Keywords:** Botswana, HOPE II, HPV—human papillomavirus, HPV vaccination, vaccination records

## Abstract

The World Health Organization (WHO) estimated 660,000 new cervical cancer cases and 350,000 deaths globally in 2022, with over 90% occurring in low- and middle-income countries (LMICs). Global elimination targets call for vaccination of >90% of girls against human papillomavirus (HPV) by age 15 by 2030. While Botswana's national policy aligns with these targets, progress monitoring is constrained by fragmented, predominantly paper-based health information systems. We describe our experience verifying individual-level HPV vaccination status among adolescent girls and young women living with HIV (AGYWLWH) in Botswana. This activity was conducted to determine eligibility for the HOPE II trial, a randomized controlled study evaluating the efficacy of a single-dose nonavalent HPV vaccine. Verification followed the national rollout of school-based HPV vaccination in 2015, targeting girls aged 9–13 years. Participants provided pre-screening consent for review of vaccination records held in school health systems, Ministry of Health (MoH) registers, and participant-held vaccination cards where available. We sought HPV vaccination records for 80 potential participants across 80 schools and 68 clinics. Only 15 participants (18.8%) had verifiable records: 2 (2.5%) retained vaccination cards, 4 (5.0%) had documentation in school records, and 9 (11.3%) had entries in MoH registers. Most school records were incomplete or missing, and several sites reported loss or disposal of records due to relocation, fire damage, or storage constraints. These findings highlight critical gaps in HPV vaccination documentation. Strengthening electronic immunization registries, standardizing record transfer, and improving data integration could enhance monitoring and support equitable progress toward cervical cancer elimination.

## Introduction

Despite the availability of effective vaccines, disparities remain in progress toward cervical cancer elimination. The World Health Organization (WHO) reports that globally there are around 660,000 new cases of cervical cancer and around 350,000 cervical cancer related deaths annually. In 2022, 90% of cases and 94% of deaths occurred in lower-middle income countries (LMICs) where treatment options are limited (1–4). Most cervical cancer cases are caused by human papillomavirus (HPV) (5). High population-level coverage with HPV vaccination is one of the most impactful interventions to achieve cervical cancer elimination. This is highlighted in the WHO's global strategy to eliminate cervical cancer that underscores the critical role of comprehensive prevention measures which include achieving 90% coverage of HPV vaccination among 9 to 14 year old girls by 2030 (4, 6, 7). Despite these clear benefits and global commitments, many sub-Saharan African (SSA) countries continue to face significant challenges in achieving optimal HPV vaccination coverage (7–9).

In many countries, HPV vaccination coverage is estimated from administrative data, but neither complete individual-level HPV vaccination records nor national registers are available to monitor coverage by age, area, and dose completion (10). This presents significant challenges to accurately monitor vaccine uptake and identify populations that remain under vaccinated (8). Consequently, innovative strategies are needed to accurately ascertain vaccination status, and coverage to optimize appropriate interventions, particularly in settings where data infrastructure is fractured or limited (9). In Botswana particularly, cervical cancer remains the most prevalent cancer among women and the Ministry of Health (MoH) National Strategy for Cervical Cancer Prevention aims to achieve the WHO cervical cancer elimination targets by 2030. Strategies to achieve elimination included vaccination of girls under the age of 15 years old which was piloted in 2013 and national scale-up of secondary screening in 2014 (11–13). Subsequently, the national HPV vaccination program was implemented in 2015 as a school-based program targeting girls aged 9–13 years with a 2-dose schedule and three-dose schedule for adolescent girls and young women living with HIV (AGYWLHIV). Vaccination registries and vaccination cards were dispatched alongside vaccines, though utilization and safeguarding of HPV vaccination records have been inconsistent.

Botswana faces significant data management challenges in its health sector driven by fragmented systems, limited interoperability, and gaps in data quality and governance. The country relies on more than 18 separate health information systems that operate largely independently with variable reliability. Limited data exchange across systems coupled with the absence of a unified national strategy for integration, constrains effective data use and analysis. As a result, frontline clinicians often lack access to complete patient information, and key health indicators remain inconsistently captured. These parallel systems also limit coherent governance and standardization across the health sector (14). In addition, the continued reliance on paper-based systems further impedes data continuity, integration and efficient patients care (15). Within this context, we describe our experience verifying individual-level HPV vaccination status for young AGYWLHIV in Botswana.

## Methods

We conducted a retrospective, descriptive vaccination verification exercise to confirm eligibility for potential participants of the Human papillomavirus One-Dose-vaccine Prevention Efficacy among Women living with HIV (HOPE-II) Study (http://ClinicalTrials.gov: NCT06436274). The HOPE II study seeks to evaluate the efficacy of single dose nonvalent HPV vaccination in AGYWLHIV in Botswana, Rwanda, and South Africa. Inclusion criteria included living with HIV, 16 years or older, sexually active in the last 6 months, have a cervix, not enrolled in other vaccine trials, and verifiable prior HPV vaccination (Table 1). The HOPE II eligibility criteria initially required confirmation of documentation of a prior single-dose of HPV vaccination. These criteria were later amended to include participant report of any prior HPV vaccination and dosing.

**Table 1:** HOPE II inclusion and exclusion criteria.

Inclusion criteria	Exclusion criteria
1. Assigned female at birth 2. Age 16 years and above on the day of signing the ICF 3. Living with HIV with confirmed test results or clinic records 4. History of receiving a single dose of an HPV vaccine before HIV diagnosis 5. Self-reported sexually active in the last 6 months 6. Lives within the study area and willing to provide updated locator information over the course of the study 7. Does not have an autoimmune, degenerative, or genetic disease 8. Does not have known advanced HIV (as per stage IV WHO clinical staging criteria for HIV) 9. No other Investigator-determined factor would limit participation in the trial 10. Has not and is not enrolled in a monoclonal, investigational vaccine, or a large quantity blood draw study 11. The participant has a cervix	1. Anyone with gross cervical abnormality on examination 2. Anyone with an allergy to vaccine components or yeast

Participants for the HOPE-II trial were recruited in HIV care clinics in and around Gaborone, Botswana. Aligned with timing of the rollout of the HPV vaccination program in Botswana, we limited prescreening to those aged 16–25 years old. Potential participants for the HOPE II trial were prescreened and consented to allow study staff to review vaccination records in order to determine eligibility. HPV vaccination data, including, the data source, date of vaccine(s) and number of doses was collected.

We used a verification approach that was conducted across Botswana drawing from vaccination cards, school health records, and MoH HPV vaccination registers [held at individual clinics and District Health Management Team (DHMT) storage facilities]. In Botswana, all schools are assigned a clinic which carries out school health interventions including immunization. The planned documentation of HPV vaccination was multilevel: all vaccinated children were to receive their HPV vaccination card after completing their vaccination schedule, schools were to document individual-level vaccination in the pupil's school health record maintained at the school, and school health nurses were to maintain an MoH register with individual-level vaccination documented.

For participants who did not have their HPV vaccination cards, the location and name of their primary school were collected and school-based records were sought from primary schools across the country where we often traveled as far as 600km from Gaborone.

## Results

Between February and April 2025, HPV vaccination records were sought for 80 potential participants. Verifiable individual-level documentation scarce. Only two (2.5%) potential participants retained personal HPV vaccination cards and school health records were identified for just four (5.0%) of the participants across 80 schools visited. Overall, the availability of HPV vaccination data was extremely limited and inconsistent across sites. Most schools had incomplete school health records or no records at all.

A notable pattern was the disparity between HPV vaccination documentation and records of other immunizations. Documentation for other booster vaccinations were more frequently available, suggesting selective gaps in recording practices rather than a complete absence of record-keeping systems.

Although 25 schools reported holding some HPV vaccination records, these were typically incomplete, fragmented, and not linkable to the individuals under review. Structural limitations in record continuity were evident: few schools issued HPV vaccination cards to students at key transition points, such as completion of primary school or transfer to junior secondary school, reducing the likelihood of record retention at the individual level.

Schools consistently attributed missing records to systemic factors, including destruction during renovations, relocation, or fire, as well as limited storage capacity leading to routine disposal of paper-based records. Taken together, these findings indicate that the primary constraint is not the absence of data *per se*, but the lack of durable, integrated, and transferable record systems capable of supporting individual-level verification.

Nine potential participants (11.3%) had vaccination documented in MoH HPV vaccination registers. Table 2 summarizes the facilities visited and data sources reviewed. Discrepancies were observed between vaccination records verifiable at schools and those documented in MoH registers maintained at the responsible clinics. Verification of vaccination status was contingent on the availability and accessibility of records across multiple sources, which were often incomplete or inconsistently maintained. These challenges in obtaining verifiable documentation prior to enrolment in the HOPE II study necessitated a revision of the eligibility criteria to permit self-reported HPV vaccination history as an acceptable method of verification.

**Table 2:** Data sources extracted across facilities for vaccination verification purposes among 80 potential HOPE II study participants.

Data source	Total number of facilities visited (148)	HPV records present (87)	Total number of records that matched participants (15)	Proportion of records matched (out of total number of records sought)
Individual vaccine cards	N/A	2	2	2.5
School health cards	80	30	4	5.0
MoH registers held at clinics and DHMTs	68	55	9	11.3

## Discussion

Our experience highlights the potential and the limitations of relying on individual-level vaccination records to ascertain HPV vaccination status in a real-world setting. While the objective was to verify prior HPV vaccination among potential participants for a trial, our findings illuminated broader issues related to data availability, recordkeeping practices, and surveillance infrastructure that have direct implications for monitoring progress toward achieving HPV vaccination targets. Record retrieval yielded vaccination data for 18.8% of the participants with a mix of documentation sources. Despite planning for HPV vaccination documentation at 3 different levels—individual HPV vaccination card, individual-level school health records and MOH vaccination registers—maintenance of records and transfer of vaccination materials were not carried out in a systematic manner. Specifically, the handover of individual vaccination cards to young women and the transfer of school health cards from primary to secondary school at primary school exit were inconsistently implemented. Frequent staff turnovers, office relocations and inadequate administrative handovers were observed both at the administrative level and among health personnel, which further exacerbated record loss. Thus, highlighting substantial gaps in complete capture of vaccination status at the individual level.

While our experience was drawn from prescreening cohorts in HIV care clinics around Gaborone, which may not be representative of the broader population or other regions of Botswana; our findings underscore substantial coordination gaps between the Ministry of Basic Education and the MoH, despite their shared mandate for school health programs. Although both ministries collect HPV vaccination data, their information systems are neither fully integrated nor consistently aligned, resulting in discordant and fragmented records. Documentation of HPV vaccination status was highly inconsistent across sources. School health records indicated HPV vaccination for 5 potential participants, while an additional 8 confirmations were identified in MoH vaccination registers; however, there was minimal overlap between these two data sources at the individual level.

Furthermore, all 30 schools that had HPV records, reported holding incomplete individual-level HPV vaccination records, suggesting that factors external to the HPV vaccination program itself, such as record management practices, infrastructure limitations, and inter-ministerial data governance arrangements, may contribute substantially to data incompleteness. This fragmentation in record-keeping constrains the accurate estimation of vaccination coverage and poses significant challenges for verifying individual vaccination histories, particularly in settings seeking to implement integrated and comprehensive health interventions (18).

Challenges related to record-keeping and coordinated data management are particularly pronounced in settings such as Botswana, where the absence of a robust electronic national immunization registry necessitates continued reliance on paper-based documentation to verify HPV vaccination status (19, 20). The challenges observed during this exercise reflect broader systemic constraints affecting vaccine delivery, monitoring, and evaluation in comparable contexts, where efforts to achieve and sustain high coverage are frequently undermined by inadequate data infrastructure and inconsistent record-management practices (21). These systemic deficiencies impede effective public health planning, as reliable and timely vaccination data are essential for designing targeted interventions, monitoring program performance, and allocating resources efficiently (22). Moreover, fragmented and incomplete records may obscure pockets of under- or non-vaccinated individuals, thereby exacerbating health inequities and undermining progress toward cervical cancer elimination goals. It should be mentioned that the manual searching approach used during this exercise could have inadvertently overlooked some records. Furthermore, the retrospective nature of record retrieval precludes assessment of causality regarding data gaps or vaccination determinants. Nevertheless, our systematic approach to verifying records provides a realistic appraisal of data availability for program monitoring and identifies concrete, actionable areas for improving data infrastructure and record-keeping practices to inform data-driven initiatives to improve HPV vaccination coverage.

### Implications and future directions

Creating centralized vaccination registries that integrate MOH registers and school-based health data could substantially improve the ascertainment of vaccination status. This approach would also enable a more precise monitoring of vaccination uptake and coverage by age, geography, and dose completion. Standardizing retention, transfer, and handover procedures for school and healthcare records particularly during transitions such as school changes, relocations or renovations could help reduce data losses. Introducing digital records with routine backups would lessen dependence on paper files and facilitate timely data access. This shift would also improve data reliability across administrative changes and increase the use of real-time data to inform programmatic efforts. In resource-limited settings, innovative approaches may be required to approximate vaccination status when records are missing. This could include triangulating caregiver reports, school records, and MOH registers, implementing prospective record capture at vaccination points with standardized digitization workflows and prioritizing the digitization of existing paper records with reliable metadata. To achieve this an interoperable individual-level record system is useful to create a seamless, secure, and up-to-date view of each person's health information across settings, which would address fragmentation and handover gaps that undermine effective health management. Investing in an electronic national immunization registry would enable accurate, timely, and scalable monitoring of HPV vaccination coverage. Piloting and evaluating digital school health record systems with standardized data elements and creating a digital vaccination registry could improve future HPV vaccination coverage monitoring. This would require an intentional engagement with policymakers, educators, clinicians, and communities to emphasize the importance of durable, accessible vaccination records as a public health priority. Addressing these data challenges will also allow identification of under-vaccinated populations and facilitate the implementation of targeted prevention strategies.

## Conclusion

Our findings reveal substantial gaps in individual-level HPV vaccination documentation within Botswana's education and health systems, largely driven by fragmented data systems, reliance on paper-based records, and inconsistent record-holding practices. These gaps leave individuals without access to essential personal health information and limit the ability of vaccination program to accurately verify population-level coverage. The development of a national immunization registry, supported by progressive digitalization of vaccination records, is therefore critical to enable systematic tracking of HPV vaccination uptake, improve data linkage across sectors, and support data-driven decision-making at all levels of the health system. Such digital solutions have the potential to strengthen the monitoring of vaccination efforts, identify pockets of under-vaccination to address persistent inequities in HPV vaccination coverage.

## Data Availability

The raw data supporting the conclusions of this article will be made available by the authors, without undue reservation.

## References

[1] Barnabas R V Brown ER Onono M Bukusi EA Njoroge B Winer RL . Single-dose HPV vaccination efficacy among adolescent girls and young women in Kenya (the KEN SHE Study): study protocol for a randomized controlled trial. Trials. (2021) 22:661. doi: 10.1186/s13063-021-05608-8 34579786 PMC8475401

[2] Barnabas R V Brown ER Onono MA Bukusi EA Njoroge B Winer RL . Efficacy of single-dose HPV vaccination among young African women. NEJM Evidence. (2022) 1:EVIDoa2100056. doi: 10.1056/EVIDoa2100056 35693874 PMC9172784

[3] Cervical cancer. Available online at: https://www.who.int/news-room/fact-sheets/detail/cervical-cancer (Accessed April 8, 2026).

[4] Cervical Cancer Elimination Initiative. Available online at: https://www.who.int/initiatives/cervical-cancer-elimination-initiative (Accessed April 8, 2026).

[5] Fast about HPV and cervical cancer | UNICEF. Available online at: https://www.unicef.org/stories/fast-facts-hpv-cervical-cancer (Accessed February 17, 2026).

[6] Avila-Aguero ML Ospina-Henao S Brenes-Chacon H Espinal-Tejada C Trejo-Varon R Morice A. Human papilloma virus vaccination as a strategy to eliminate cervical cancer: challenges and opportunities. Vaccines. (2025) 13:297. doi: 10.3390/vaccines13030297 40266227 PMC11945881

[7] Denny L Saidu R Boa R Mbatani N Castor D Moodley J . Point-of-care testing with Xpert HPV for single-visit, screen-and-treat for cervical cancer prevention: a demonstration study in South Africa. Sci Rep. (2023) 13:16182. doi: 10.1038/s41598-023-43467-2 37758811 PMC10533854

[8] Delany-Moretlwe S Kelley KF James S Scorgie F Subedar H Dlamini NR . Human papillomavirus vaccine introduction in south africa: implementation lessons from an evaluation of the national school-based vaccination campaign. Glob Health Sci Pract. (2018) 6:428–38. doi: 10.9745/GHSP-D-18-00090 30143561 PMC6172125

[9] Sibomana H Ukwishaka J Mtenga H Luoga O Acosta D Fisher-Borne M . Assessing users' experiences and integration of digital health interventions for Human Papilloma Virus vaccination and cervical cancer services in Rwanda. BMC Digit Health. (2024). doi: 10.21203/rs.3.rs-4741668/v1

[10] Bruni L Saura-Lázaro A Montoliu A Brotons M Alemany L Diallo MS . HPV vaccination introduction worldwide and WHO and UNICEF estimates of national HPV immunization coverage 2010–2019. Prev Med. (2021) 144:106399. doi: 10.1016/j.ypmed.2020.106399 33388322

[11] National training boosts cervical cancer prevention efforts in Botswana | WHO | Regional Office for Africa. Available online at: https://www.afro.who.int/countries/botswana/news/national-training-boosts-cervical-cancer-prevention-efforts-botswana (Accessed February 17, 2026).

[12] DiAngi YT Panozzo CA Ramogola-Masire D Steenhoff AP Brewer NT A. Cross-sectional study of HPV vaccine acceptability in Gaborone, Botswana. PLoS ONE. (2011) 6:e25481. doi: 10.1371/journal.pone.0025481 22039413 PMC3201944

[13] Grover S Raesima M Bvochora-Nsingo M Chiyapo SP Balang D Tapela N . Cervical cancer in Botswana: current state and future steps for screening and treatment programs. Front Oncol Front Res Found. (2015) 5:239. doi: 10.3389/fonc.2015.00239 26579491 PMC4630577

[14] Faulkenberry G Masizana A Mosesane B Ndlovu K. Clinical data flow in botswana clinics: protocol for a mixed-methods assessment. JMIR Res Protoc. (2024) 13:e52411. doi: 10.2196/52411 39383523 PMC11514319

[15] Seitio-Kgokgwe O Gauld RDC Hill PC Barnett P. Development of the National health information systems in Botswana: pitfalls, prospects and lessons. Online J Public Health Inform. (2015) 7:e210–e210. doi: 10.5210/ojphi.v7i2.5630 26392841 PMC4576446

